# Non-Human Primate: An Essential Building Brick in the Discovery of the Subthalamic Deep Brain Stimulation Therapy

**DOI:** 10.3389/fnagi.2015.00252

**Published:** 2016-01-12

**Authors:** Abdelhamid Benazzouz, Christian Gross, Bernard Bioulac

**Affiliations:** ^1^Univ. de Bordeaux, Institut des Maladies Neurodégénératives, UMR 5293Bordeaux, France; ^2^Centre National de la Recherche Scientifique, Institut des Maladies Neurodégénératives, UMR 5293Bordeaux, France

**Keywords:** Parkinson disease, deep brain stimulation (DBS), subthalamic nucleus, functional neurosurgery, Parkinsonian patients

The recent announcement that a Lasker award has been attributed to Professor Alim-Louis Benabid for pioneering the application of high frequency stimulation, named deep brain stimulation, of the subthalamic nucleus in patients suffering from Parkinson's disease prompts us to evoke the decisive role played by the non-human primate model in the discovery of this neurosurgical therapy, now regarded as the current therapeutic gold standard of the disease.

The era of human functional surgery started in the 1950s by performing ablative procedures aiming to avoid the side effects of the commonly performed frontal lobotomy in the treatment of psychiatric illness (Spiegel et al., 1952). During surgery, intraoperative electrical stimulation was used from the beginning to explore the brain target prior to ablation. During the same period of time, Delgado et al. (1952) were the first to develop a technique of subcortical stimulation using chronically implanted electrodes connected to a subcutaneous receiver implanted in the scalp, a “Stimoceiver,” that could be controlled by radio waves. This technique of “radio communication with the brain” was initially developed for use in psychiatric patients. The chronological order of applications of chronic deep brain stimulation was first for psychiatry and behavior, then for pain and epilepsy. Deep brain stimulation for Parkinson's disease and other movement disorders was the last indication of the older era of chronic subcortical stimulation. Hariz and colleagues reported a detailed history of deep brain stimulation between 1947 and 1987 (Hariz et al., 2010).

In the late 1980s, considerable progress was made in the understanding of the role played by the basal ganglia in the pathophysiology of Parkinson's disease (Albin et al., 1989) and the subthalamic nucleus was highlighted as an optimal therapeutic target. This small nucleus, the only excitatory glutamatergic structure of the basal ganglia network (Smith and Parent, 1988) playing a key role in movement control (DeLong, 1990), is considered as a driving force regulating the basal ganglia output nuclei; it was therefore suggested that activation of the subthalamic nucleus should inhibit movement and conversely its inhibition should be related to the release of movement. Back in 1927 it had already been reported that a focal lesion of the subthalamic area could induce hemiballismus, the manifestation of abnormal involuntary movements (Martin, 1927) and this association was reproduced later by lesion of the subthalamic nucleus in the non-human primate (Hammond et al., 1979). Single unit extracellular electrophysiology (Miller and De Long, 1987) and autoradiography of 2-deoxyglucose uptake (Mitchell et al., 1989) likewise showed that systemic injection of the neurotoxin 1-methyl-4-phenyl-1,2,3,6-tetrahydropyridine (MPTP) in monkeys resulted in dopamine cell degeneration in the substantia nigra *pars compacta* and the manifestation of akinesia and rigidity, associated with a hyperactivity of subthalamic nucleus neurons.

In the light of these results, Bergman et al. (1990) and Aziz et al. (1991) a year later proposed therapeutic lesion of the subthalamic nucleus and both demonstrated an effective alleviation of parkinsonian motor symptoms in monkeys. Since lesioned animals unfortunately also developed severely abnormal involuntary movements, this irreversible approach could not be proposed to patients.

Pursuing a parallel line of research, our team in Bordeaux at the “*Centre National de la Recherche Scientifique*” (CNRS) was the first, in the period 1991-1992, to carry out rigorous studies of the effects of high frequency stimulation of the subthalamic nucleus in Macaca mulatta monkeys rendered parkinsonian by MPTP, observations which allowed objective quantification of the modifications of the principal movement parameters this treatment induced. The results obtained were spectacular; rigidity disappeared and akinesia was hardly observable (Benazzouz et al., 1993).

Impressed by these results, we proposed the transfer of this neurosurgical approach to parkinsonian patients. Only a few months later, in collaboration with our team, Alim-Louis Benabid who was already a recognized expert in the field of functional neurosurgery and who realized the importance of these observations, attempted the first application of high frequency stimulation of the subthalamic nucleus in human patients (Pollak et al., 1993; Limousin et al., 1995) with a success that has never failed since.

This example of cooperation provides a perfect illustration of how irreplaceable the phylogenetic proximity between humans and non-human primates can be, not only from the physiological and clinical point of view but also for the design of relevant pathophysiological models and the definition of new therapeutic strategies.

In such an exemplary case of ≪ bench to bedside ≫ translation, it is important to cite all the links that have built the chain and engendered such positive results.

This is a new era of neuromodulation and it is clear now that the success of this functional neurosurgery involves the modulation of circuitry properties and cortical-subcortical synchronies involving also stations not strictly attributable to the direct/indirect pathway scheme (Stefani et al., 2007). Furthermore, despite possible adverse events, deep brain stimulation in patients contributed in large part to the understanding of the pathophysiology of Parkinson's disease and opened the way to the treatment of a number of chronic and disabling neurologic and psychiatric disorders. In order to ensure continued progress in this field, we need to have a better understanding of the pathophysiology of brain diseases, as well as to develop a next generation of electrodes and stimulation devices with combined electrical and chemical sensing (Rosin et al., 2011; Priori et al., 2013). These technological improvements cannot be done without complimentary works in patients and laboratoty animal models of brain diseases.

## Funding

This study was supported by the “Centre National de la Recherche Scientifique and Université de Bordeaux.”

### Conflict of interest statement

The authors declare that the research was conducted in the absence of any commercial or financial relationships that could be construed as a potential conflict of interest.
